# EXPLORING THE IMPACT OF CHILDHOOD TRAUMA, SOCIAL NETWORK, AND GENDER MODERATION ON DEPRESSION AMONG OLDER ADULTS

**DOI:** 10.1093/geroni/igae098.2674

**Published:** 2024-12-31

**Authors:** Ying Xu, Xiaoyu Fu, Merril Silverstein

**Affiliations:** Syracuse University, Syracuse, New York, United States; Syracuse University, Syracuse, New York, United States; Syracuse University, Syracuse, New York, United States

## Abstract

China’s aging population faces significant challenges, including a high prevalence of depression among older adults in rural areas (Li et al., 2021). This study investigates the impacts of childhood trauma and social network on depression among older adults and whether their impacts vary by gender in rural China. Using data from the 2021 wave of the Longitudinal Study of Older Adults in Anhui Province, our sample comprises 1558 participants, including 812 males and 746 females. Hierarchical moderated regression models were employed, controlling for age, marital status, living conditions, and education. Results revealed that childhood trauma significantly associated with older adults’ depression level; however, its impact was notably stronger for females than males, as indicated by the significant trauma x gender interaction (β = 0.048, p <.05). Negative main effect of social network on depression was found (β = -0.148, p <.05), suggesting that older adults with larger social network tend to have lower level of depression regardless of their gender. The findings underscore the need for gender-specific approaches to addressing trauma-related depression among older adults. Tailored interventions and supports, particularly for females who have experienced trauma, are warranted. Moreover, programs aimed at enhancing the quality of older adults’ social networks may help reduce depression in both genders.

